# Gene Expression Profile in the Liver of Sheep Infected with Cystic Echinococcosis

**DOI:** 10.1371/journal.pone.0160000

**Published:** 2016-07-28

**Authors:** Wenqiao Hui, Song Jiang, Xianxia Liu, Qian Ban, Sheng Chen, Bin Jia

**Affiliations:** 1 Institute of Animal Husbandary and Veterinary Medicine, Anhui Academy of Agriculture Sciences, Road Nongkenan, Hefei, 230031, Anhui, People’s Republic of China; 2 College of Animal Science and Technology, Shihezi University, Road Beisi, Shihezi, 832003, Xinjiang, People’s Republic of China; 3 Center for Stem Cell and Translational Medicine, School of Life Sciences, Anhui University, Road Jiulong, Hefei, 230000, Anhui, People’s Republic of China; Second University of Naples, ITALY

## Abstract

**Background:**

Cystic Echinococcosis (CE), caused by infection with the *Echinococcus granulosus* (*E*. *granulosus*), represents considerable health problems in both humans and livestock. Nevertheless, the genetic program that regulates the host response to *E*. *granulosus* infection is largely unknown. Previously, using microarray analysis, we found that the innate immunity played a vital role in the *E*. *granulosus* defense of the intestine tissue where *E*. *granulosus* first invaded. Subsequently, we turned our attention to investigating the molecular immune mechanism in its organ target, the liver, which is where the *E*. *granulosus* metacestodes are established and live for very long periods. In this work, the microarray-based methodology was used to study gene expression profiles in the liver of sheep infected with *E*. *granulosus at* 8 weeks post infection, corresponding to the early cystic established phase.

**Methods:**

A total of 6 female-1-year-old healthy Kazakh sheep were used for the experiments. Three Kazakh sheep were orally infected with *E*. *granulosus* eggs, and the others remained untreated and served as controls. Sheep were humanely euthanized and necropsized at 8 weeks post-infection (the early stage of cyst established). The microarray was used to detect differential hepatic gene expression between CE infection sheep and healthy controls at this time point. Real-time PCR was used to validate the microarray data.

**Results:**

We found that *E*. *granulosus* infection induces 153 differentially expressed genes in the livers of infected sheep compared with healthy controls. Among them, 87 genes were up-regulated, and 66 genes were notably down-regulated. Functional analysis showed that these genes were associated with three major functional categories: (a) metabolism, (b) the immune system and (c) signaling and transport. Deeper analysis indicated that complement together with other genes associated with metabolism, played important roles in the defense of *E*. *granulosus* infection.

**Conclusion:**

The present study identified genes profiling in the liver tissue of *E*. *granulosus* infection in sheep. The expression pattern obtained here could be helpful for understanding the molecular immunity mechanisms of host responses to *E*. *granulosus* infection. However, it is necessary to carry out further studies to evalute the role of these genes.

## Introduction

Echinococcosis is one of the most geographically widely distributed parasitic zoonosis. It is caused by infection with the larval stage of the cestode *Echinococcus*, including *Echinococcus granulosus*(*E*. *granulosus*) and *Echinococcus multilocularis*(*E*. *multilocularis*), and the both species are responsible for the disease, which mainly affects human and animals [1]. *E*. *granulosus* infection occurs in the intermediate host (human and domestic animals, like sheep, camel, cow, et al.,), by a series of successive events: when ingested by intermediate hosts, the *E*. *granulosus* eggs hatched in the intestine will transform into oncosphere phase with the help of bile juice, which penetrate through the intestinal wall, and eventually reside in the internal organs by following the portal blood stream. Most commonly, primary infection develop in the liver [2–4], where the oncosphere metamorphoses into the next larval stage, the metacestode, which could overcome the immune system and subsequently develop as fluid-filled cysts in the liver, leading to mechanic pressure and to pathological changes associated with compression or obstruction [1,4].The disease, also called cystic echinococcosis (CE), is usually prevalent in pastoral and/or semipastoral area in China, Central Asia, Middle East, South America, and some part of Europe. CE not only contributes to be a major public health issue in areas of poor sanitary and hygiene, but also makes herdsman suffering from economic losses due to animal health problems in many rural areas of the world [1,5,6].

For many years, several efforts have been made to control CE infection. One of the most appealing strategies for such prevention is the immunology study, as a better understanding of the immune events during the infection process is extremely of importance in developing immunodiagnostic kits and highly effective recombinant vaccines against *E*. *granulosus* infection. In succession, a multitude of excellent reports on the immunology of echinococcosis have been published out, on the theme of highlighting variability in immunological responses between individuals including high or low antibody responders and Th2- or Th1-dominant cytokine profiles [7–18]. Among them, most studies carried out were either upon *in vitro* experiment (culturing cyst) [9,10,14] or so-called secondary infections (intraperitoneal inoculation of fully developed metacestode cyst) [11,13,18]. To fully understand the biology events of CE, it is, therefore, necessary to carry out a primary infection experiment, by perorally inoculation of infectious *E*. *granulosus* eggs in animals, resulting in an intrahepatic cysts growth of the metacestode that overcomes the immune system and subsequently established a chronic phase of infection.

With the character of highly susceptible to CE, sheep is an excellent model to study the host-parasite interplay [19]. Previously, by orally infected with *E*. *granulosus* eggs, sheep are primary infection with CE, and the microarray analysis on gene expression profiles in the intestine of sheep were carried out by us. We found that the innate immunity response was activated in the parasite locating intestine stage of infection, which reflected the molecular immunological mechanism of early infection to some extent [20]. However, since parasite cysts are able to live for very long periods in the infected intermediated host, it is, therefore necessary to understand the mechanisms that *E*. *granulosus* evades or modulates the host immune response through. To date, the genetic program that regulates the mechanisms by which *E*. *granulosus* infection occurs and induces liver pathology is largely unknown, although the liver is most frequent location of echinococcal cysts in the liver, representing approximately 70% of cases echinococcal cysts [2,3]. In turn, studies on the molecular mechanism of host response to AE caused by infection with *E*. *multilocularis* have been well documented in the hepatic transcriptome level [21–23], and furthermore, the nuclear genomes of *E*. *multilocularis* and *E*. *granulosus* have already been characterized recently [24,25], which may help gaining a deeper understanding of host-parasite interaction [26].

In the present study, to further understand the host defense mechanisms and identify effector mechanisms of host response to *E*. *granulosus* infection, we examine changes in gene expression in the livers of sheep infected with *E*. *granulosus*, by using microarray-based methodology.

## Materials and Methods

### Ethics statement

All animals were raised and handled in strict accordance with the Animal Ethics Procedures and Guidelines of the People’s Republic of China. The protocol was approved by the Institutional Animal Care and Use Committee of Shihezi University.

### Experimental animals, parasites, and infections

An established sheep model of primary cystic echinococcosis was used as previously described [20]. Briefly, one-year-old female healthy Kazakh sheep purchased from the No.165 farm, (Tacheng, Xinjiang) and were raised in parasite-free conditions of Shihezi University. They were randomly allocated into infection (n = 3) and control groups (n = 3). They were negative for antibodies to hydatid cyst fluid (HCF) antigen, assayed by a commercial ovine hydatidosis ELISA kit (Shenzhen Combined Biotech Co., Shenzhen, China), and no hydatid cysts presented in internal organs detected by ultrasonography prior to the experiment.

Following strict safety, three sheep were orally infected with 5000 *E*. *granulosus* eggs and the other three were kept as uninfected controls. The parasite eggs isolation and safety handling were described in our previous study [20]. After infection, the ultrasound was performed to detect the parasitic lesions or hydatid cyst on the liver per week. At 8 weeks (8wks) post-infection, animals were sacrificed with an overdose of euthanasia medicine containing hydroxybutyramide, methylene ammonium iodide, tetracaine (100mg/kg, IV route) for the healthy control group and for the group representing the chronic stage of primary CE.

The infection of CE in the liver was detected by counting the number of parasitic lesions macroscopically visible on and within the liver tissue. The tissues selected methods in this study was according to the references reported by Gottstein et al. [21] and Wang et al. [23]. In *E*. *granulosus* infected sheep, the liver tissue was removed and approximately 100 mm^3^-sized periparasitic liver tissue blocks (close to the lesion by 5mm, avoiding the contamination by parasitic *E*. *granulosus* tissue/cells and correspondingly involved infiltrating host immune cells) were dissected, while control samples in healthy sheep were taken from the same liver lobe. Tissue blocks were directly deep-frozen in liquid nitrogen prior to long-term storage at -80°Cuntil RNA extraction.

### RNA reparation, Labeled cRNA preparation and microarray processing

Total RNA was isolated from liver tissues using TRIzol reagent (Invitrogen, USA), according to the manufacturer’s instructions. Isolated RNA was purified by RNeasy Mini Kit (Qiagen, Germany). RNA quality was checked by spectrophotometric analysis (NanoDrop Technologies, Thermo Scientific, USA), and electrophoresis prior to cDNA synthesis.

The first strand cDNA was synthesized, followed by second strand cDNA synthesis. cDNA was then transcribed and labeled with T7 RNA Polymerase and cyanine 3-CTP. After purified by RNeasy Mini Kit (Qiagen, Germany), the labeled cRNA was fragmented to segments using fragmentation buffer at 60°C for 30 min. Then, the fragmented cRNA were then hybridized to Agilent custom 4×44k chips, representing 15008 sheep genes, provided by Beijing Protein Innovation Company. Hybridization, staining, and washing of all arrays were performed at Beijing Protein Innovation Co., Ltd.

### Real-time PCR validation

Quantitative real-time PCR was employed to verify the regulation of genes detected by microarray. Six differential expression genes were randomly selected as quantitative real-time PCR analysis. The GAPDH was used as an internal control. The primers for these genes, listed in Table 1, were designed by Primer premier 5.0 and synthesized by Beijing BGI Company. RNA isolation was followed by DNase treatment (Tiangen, China) to remove genomic DNA contamination. The RNA was then used for first cDNA synthesis. Aliquots of cDNA target template were diluted serially and mixed with 200nM primers. The reaction was carried out in a PCR master mix containing 12.5μl SYBR^®^ Premix Ex, 0.5μl each primer (10μ mol/L), 2μl cDNA and 4.5μl ddH_2_O in a total volume of 20μl. The PCR reactions were carried out and the cycling program was set as follows: an initial denaturing step at 94°C for 4min, followed by 35 cycles of 94°C for 15s, annealing at 52–64°C for 20s, and extension at 72°C for 20s, and a final extension step of 10 min at 72°C. Amplification reactions in triplicate for each sample were performed. The relative quantification of the target mRNAs was carried out using the comparative method according to the instruction manual. The mRNA expression levels for all samples were normalized to the level for GAPDH housekeeping genes.

**Table 1:** Primer sequence for qRT-PCR analysis of gene transcripts.

Gene	Primer sequences (5’-3’)	Annealing temperature(°C)	Expected size (bp)
GAPDH	F:CTGACCTGCCGCCTGGAGAAA	59.0	149
	R: GTAGAAGAGTGAGTGTCGCTGTT		
NDUFA1	F: AAGGGACCTGGAAGGGAGT	57.0	176
	R: CTGATATGAATAATGGGCAACC		
APOA4	F: GAGCCGAGGCGGAGGTCAAT	64.0	237
	R: CGGAGTCCTTAGTCAGCCGTTCAT		
CⅡTA	F: CAAAGCATGACCGCTGGAAATT	57.0	209
	R: AAACAAACAGGAAATGGAGGCAAA		
SLC7A11	F: ACCCTTTGACAATGATAATGC	54.0	111
	R: GATAAATCAGCCCAGCAACT		
C6	F: CAACATCCAGCCATCACTT	54.0	245
	R: GGAGGGTAACAGGCAGACAC		
PI4K2B	F: GAAGAGGGTCCGCAGTTA	52.0	185
	R: TTGACGCAGAAGAGTTGG		

### Data filtering and statistical analysis

The hybridization data were extracted with Feature Extraction Software 10.7. Gene Spring 12.0 Software was used for data analysis. The read dates were normalized. For each microarray experiment, a Student *t* test with a false discovery rate (FDR) of 0.05 was amplified to test the hypothesis that a gene’s expression does not differ between infected and control sheep. Statistically, only genes with a Student’s t test *P*-value<0.05 and a fold change(FC)≥2 were selected as differential expression ones.

## Results

In this study, eight weeks after infection, all of infected sheep had cystic echinococcosis in the liver as evidenced by the presence of hepatic liver lesions, exhibiting the same morphology including a central parasitic vesicle of approximately 0.2–0.5 cm of diameter, and surrounded by a white periparasitic inflammatory corona (S1 Fig). The control counterparts neither presented macroscopically nor microscopically visible lesions in the liver.

In the present study, we analyzed gene expression in the liver of sheep infected with *E*. *granulosus* and compare it to that for uninfected sheep. Samples were collected at 8wks post infection, which corresponds to the early metacestode cyst established phase in the liver. Microarray analysis displayed that *E*. *granulosus* infection induced significant changes regarding the hepatic gene expression profile in affected sheep compared with the untreated controls. The parasitized animals shared a total of 153 genes that significantly changed after 8 wks of infection (Table 1). Of those genes, 87 appeared were up-regulated in reference to non-infected controls, and 66 genes were notably down-regulated. We manually assigned each of these transcripts to functional categories, with a significant focus on genes associated with three major functional categories: (a) metabolism, (b) the immune system and (c) signaling and transport. Table 2 listed information of some up- or down-regulated differentially expressed genes of different canonical pathways in the liver infected with *E*. *granulosus* at 8 wks post infection, compared with healthy controls.

**Table 2:** Partial differential expression genes in the liver, organized according to their function groups.

Gene	Accession number	Gene ID	Description	FC
**Genes associated with Metabolism**				
*Genes associated with mitochondrial respiratory chain*				
NDUFA1	NM_001318974.1	974987257	NADH dehydrogenase 1	62.2
SOD1	NM_001145185.1	223633903	superoxide dismutase 1, soluble	2.67
AACS	XM_012097667.2	965982956	acetoacetyl-CoA synthetase	17.4
P450RAI-2	NM_007811.2	178057350	cytochrome P450 retinoid metabolizing protein	16.2
MRPL39	XM_004002812.3	965916887	mitochondrial ribsomal protein L39, nuclear gene encoding mitochondrial protein	7.54
MCM	XM_004014349.3	965967846	methylmalonyl CoA mutase	80.8
DARS	NM_001161887.1	240849240	aspartyl-tRNAsynthetase	12.6
*Genes associated with lipid metabolism*				
SCD	NM_001009254.1	57164288	stearoyl-CoA desaturase (delta-9-desaturase)	35.8
FASN	XM_015098375.1	965957117	fatty acid synthase	16.3
THRSP	XM_004019431.3	965995379	thyroid hormone responsive	19.9
HMGCS1	XM_015101308.1	965980758	3-hydroxy-3-methylglutaryl-Coenzyme A synthase 1	14.3
SQLE	NM_001104929.1	157278599	squaleneepoxidase	12.1
FDPS	XM_004002589.2	802989579	farnesyldiphosphate synthase	16.5
SCAP	XM_015102452.1	965990456	SREBF chaperone	-14.6
PLA2G2A	XM_012153325.2	965923174	phospholipase A2, group IIA	-61.7
APOA1	XM_012095497.1	803156056	apolipoprotein A-I	-2.9
APOA4	XM_004016047.3	965976075	apolipoprotein A-IV	-29.8
CES8	BC166638.1	187252608	carboxylesterase 8	-35.4
*Genes associated with blood metabolism*				
HBB	NM_001097648.1	164448673	hemoglobin-beta	37.0
HBG	AH001245.1	165904	hemoglobin-gamma	47.9
HP	NM_001040470.2	402743675	Haptoglobin	-41.0
ACE2	XM_012106267.2	966008903	angiotensin I converting enzyme (peptidyl-dipeptidase A) 2	10.4
Mllt1	XM_015095867.1	965936538	myeloid/lymphoid or mixed-lineage leukemia 1	14.0
HIG2	GAAI01005551.1	509131589	hypoxia-inducible protein 2	-9.6
THBS4	XM_015096703.1	965943888	thrombospondin 4	-14.4
BLVRA	XM_004007991.2	803063619	biliverdinreductase A。	-10.7
*Genes associated with muscle contraction*				
SNTB1	XM_015097677.1	965952304	Basic beta 1 syntrophin-like	25.2
Gabrb2	JQ911755.1	387158479	Gamma-aminobutyric acid (GABA) A receptor, subunit beta 2	12.1
Pou2af1	XM_004016007.3	965976872	POU class 2 associating factor 1	13.5
**Genes associated with signaling and transport**				
SLC7A11	NM_001252182.1	356582217	solute carrier family 7, member 11	24.4
SLC1A2	XM_012096232.2	965978842	solute carrier family 1, member 2	228.6
CACNB3	XM_012174365.2	965926784	calcium channel, voltage-dependent, beta 3 subunit	-2.8
SLC16A6	XM_015098988.1	965962049	solute carrier family 16, member 6	-19.6
SLC13A5	XM_004012581.3	965958686	solute carrier family 13, member 5	-12.5
**Genes associated with Immune response**				
*Complement*				
C6	XM_004017016.3	965980802	complement component 6	2.9
C4A	XM_012100915.1	803188285	complement component 4A	2.3
CR2	NM_001009724.1	57526243	complement component (3d/Epstein Barr virus) receptor 2 (CR2)	4.8
C3	XM_015095649.1	965934749	complement component 3	4.5
LYSB	XM_004014566.3	965968945	intestinal lysozyme	-112.7
LBP	NM_001135930.1	209693409	Lipopolysaccharide binding protein	2.2
ULBP27	NM_001168616.1	274317279	UL16-binding protein 27	54.7
*Antigen processing cell*				
MHC-DQA2	Y07898.1	1542854	MHC class II,DQA 2	-2.3
CD3D	NM_001009382.1	57164384	CD3d molecule, delta	-2.9
CD1	Z36890.1	1296950	CD1	-3.3
CD4	NM_001129902.1	194018571	CD4	-2.6
SWAP70	XM_004016163.2	803156750	SWAP switching B-cell complex 70kDa subunit	-2.1
FKBP1B	XM_015094249.1	965924814	FK506 binding protein 1B	-19.7
CD28	NM_001009441.1	57164228	CD28 molecule	-6.9
C IITA	XM_015104076.1	966002573	MHC class II transactivator	-34.4
*Antibody*				
AMICA1	GAAI01003726	509133602	adhesion molecule, interacts with CXADR antigen 1	-125.8
IGSF3	XM_015092200.1	965915911	immunoglobulin superfamily, member 3	-4.3
TMIGD1	XM_004012526.3	965958386	transmembrane and immunoglobulin domain containing 1	9.3
IGSF11	XM_015092456.1	965917273	immunoglobulin superfamily, member 11	11.7
IGSF5	XM_004003353.3	965918454	immunoglobulin superfamily, member 5	-16.2
*Cytokines*				
CXCL2	GAAI01002159	509135527	chemokine (C-X-C motif) ligand 2	13.0
CCR6	XM_015097525.1	965951574	chemokine (C-C motif) receptor 6	-5.6
CXCR6	XM_004018516.3	965990585	chemokine (C-X-C motif) receptor 6	-2.7
**Genes associated with liver injury**				
**Apoptosis**				
PI4K2B	XM_004009745.3	965941690	phosphatidylinositol 4-kinase type 2 beta	10.5
FAIM2	XM_015094686.1	965926736	Fas apoptotic inhibitory molecule 2	65.76
P21	XM_012107531.2	966011943	p21 protein (Cdc42/Rac)-activated kinase 1	2.22

Note: Table 2 shows partially differential expression genes (*E*. *granulosus* challenged at 8 wks *vs*. Healthy controls) with *P*-value<0.05 and fold change (FC)≥2, which were listed with their respective function.

At 8wks post infection, the most prominent genes which were significantly up-regulated in the liver tissue encode MCM, NDUF1, AACS, P450RAI-2, DARS, MRPL39, SOD1, associated with mitochondrial respiration and aerobic metabolism (Table 2). Consistent with the up-regulation of genes associated with mitochondrial energy metabolism, we also observed several represented up-regulated genes involved in the lipid metabolism, mainly on the aspect of fatty acid synthesis (e.g. FASN, SCD) and cholesterol synthesis, like HMGCS1 and THRSP. Instead of up-regulation, genes encoding apolipoprotein, including APOA1 and APOA4, were observed down-regulated in the present study (Table 2).

We also observed a significant increase of hemoglobin-related genes mRNA expression in infected animals at 8wks post infection, including hemoglobin-related subunitsβ(hbβ),γ(hbγ). Furthermore, the up-regulation of ACE2 and Mllt1, as well as the down-regulation of HIG2, THBS4, and BLVRA predicted to be involved in blood metabolism, was detected in infected livers, respectively. In addition to genes associated with vasodilation, genes associated with muscle contraction were also observed that show high expression profiles, they were SNTB1, Gabrb2, and Pou2af1 (Table 2).

In the present study, we observed an increase in the gene expression for complement including C6, C4A, CR2, C3 at 8wks post infection. In contrast to up-regulation, here, we observed a strong down-regulation of genes that are associated with antigen presenting cells (MHC, CIITA, CD1, CD3, CD4, CD28), antibody (IGSF3, IGSF5) in CE infection sheep at 8wks after the experimental challenge (Table 2). We note additional key changes of genes associated with transport and signaling, e.g.SLC1A2, CLIC2, CACNB3, etc.

**Fig 1:**
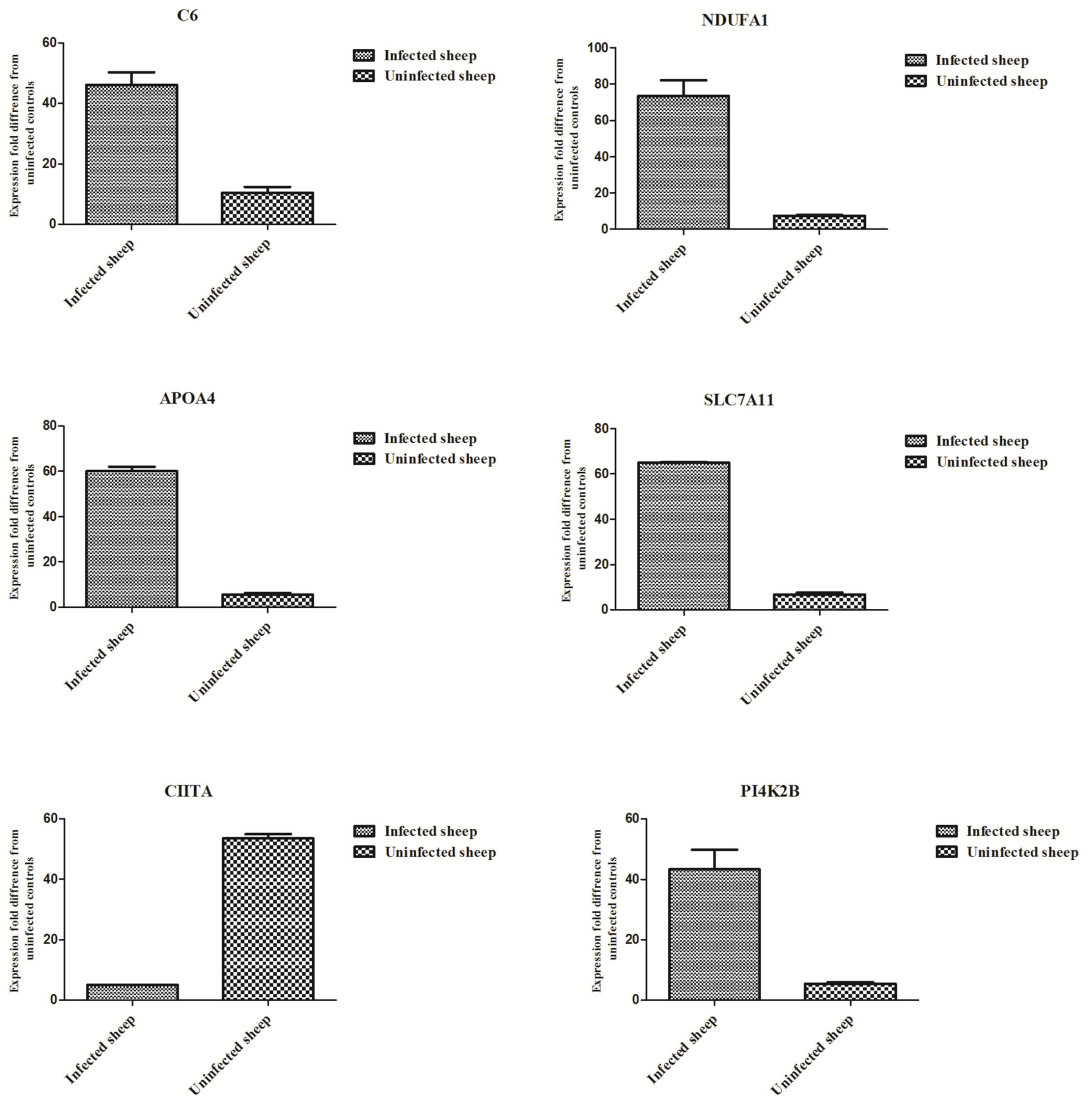
Validation of microarray data by qPCR on six randomly selected genes.

Six genes with a differential expression level were randomly selected to conduct Real-time PCR validation of microarray data. The results from Real-time PCR were shown in Fig 1, which were highly correlated with those generated from microarray analysis.

## Discussion

The immune mechanisms responsible for elimination of *E*. *granulosus* from the infected host and those may contribute to pathology during infection have not been clearly defined. The present report is a continuation of our studies to understand fundamental molecular mechanism of response to cystic echinococcosis in sheep. In the present study, we aimed to examine details about signaling pathways and key signaling molecules involved in liver defense mechanisms against *E*. *granulosus*. To our knowledge, this is the first report describing the transcriptional response of liver to *E*. *granulosus* at 8wks post infection. The time point is selected due to the reason that the stage of 8wks post infection with *E*. *granulosus* corresponds to the early metacestode cyst established phase in the liver [27]. It has been demonstrated that larval parasites, at the early stages of infection, were more susceptible to immunological killing [28]. Furthermore, in AE infection mice, it has been reported that during 1-2months infection, the hepatocyte proliferation occurred, and might play an important role in fighting against parasites [23]. Therefore, we hypothesized that critical host immune response might occurred at this time, and we expected that effector mechanisms responsible for elimination of the *E*. *granulosus* to be apparent at this stage in sheep with CE infection.

**Fig 2:**
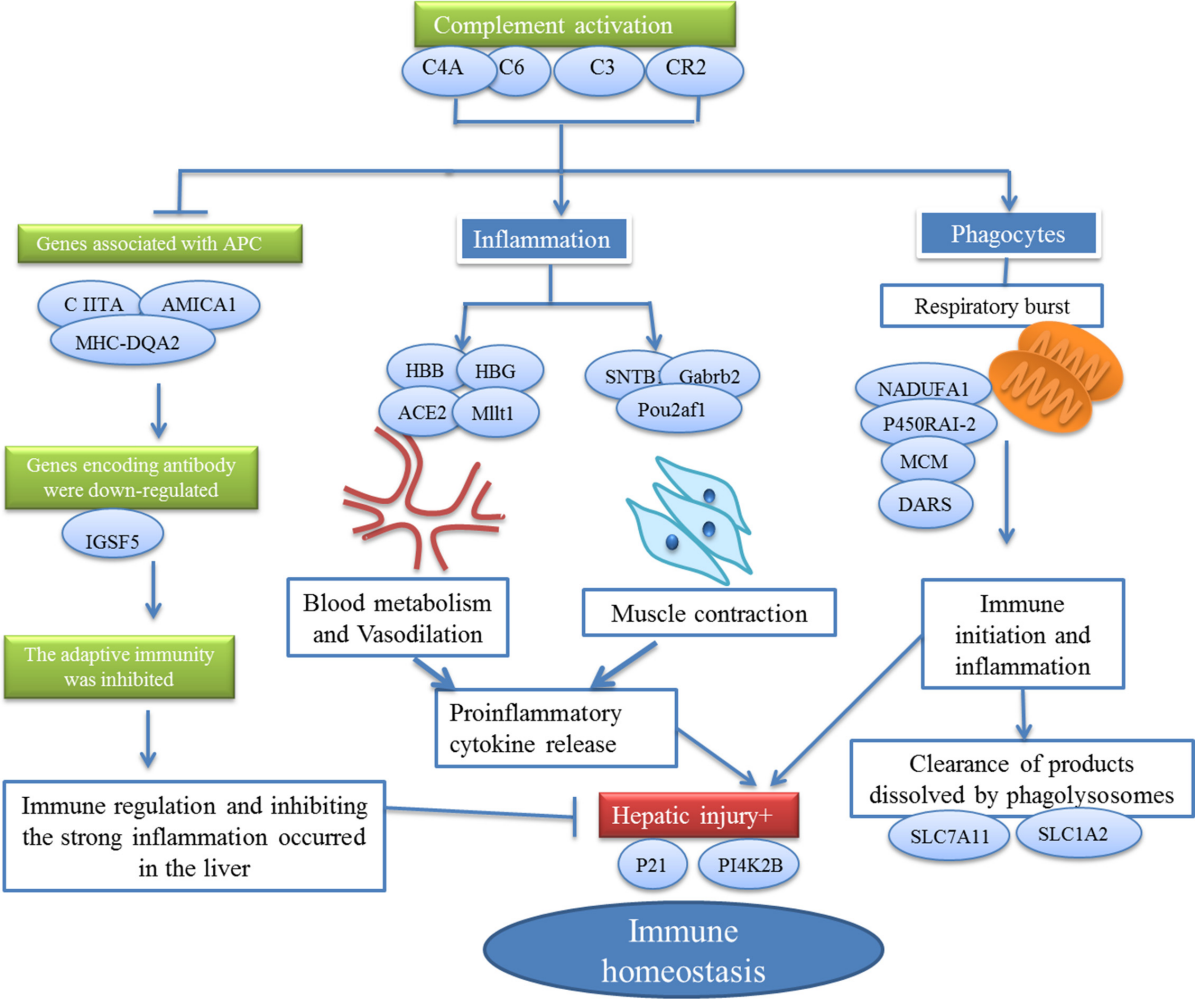
Schematic diagram summarizing interaction linked to differential expression genes in the liver of sheep infected with CE.

Actually, by applying microarray technology to *E*. *granulosus* infection, we have identified 153 genes whose expression level either increased or decreased more than 2-fold. In assessing the inferred function of these genes, we see a number of key themes, with a significant focus on genes associated with three major functional categories: a) metabolism; b) immune response; c) transport. Through a deeper analysis, we found that our data not only was suggestive of an up-regulation of many individual genes involved in immunity and metabolism systems, but also was indicative of a hub-like network tightly connected to each other, acting together to play the role of fighting against *E*. *granulosus* infection: The immune system, especially the complement, tightly connected to other systems, acting together to play the immune surveillance role during the *E*. *granulosus* infection (Fig 2).

It has been traditionally acknowledged that the complement system is a vital part of immune responses, which plays a major role in defence against the establishment of invading pathogens [29]. The complement activation triggers significant host defence mechanisms, such as phagocytosis, opsonisation and cytolysis resulted from foreign cells including pathogens, as well as the formation of membrane-attack complexes. [30,31]. In this study, *E*. *granulosus* infection at 8wks resulted in genes encoding complements activated. Combined with the alteration of genes associated with metabolism, and other systems, we summarized that the killing of parasite by activated complement might be the result from the following processes: (i) Complement-mediated early inflammation; (ii) Complement mediated phagocytosis. Fig 2 demonstrated the schematic interaction linked to differential expression genes.

To begin with, complement-mediated early inflammation may play an effective way in the parasite defense at 8wks post infection, as suggested by the alteration of genes associated with inflammatory processes, including complements, inflammatory cytokines secretion, vasoconstriction/vasodilation, muscle contraction, as well as blood metabolism.

In the present study, we observed expression profiles of genes associated with inflammation were significantly up-regulated, including HBB, HBG, ACE2; SNTB1, Gabrb2, Pou2af1, CXCL2, etc. There might be several reasons behind this phenomenon, which were illustrated in Fig 2. Microarray analysis displayed that numerous genes associated with complement pathways were induced following infection. We note expression of genes associated with complements and chemokines were significantly up-regulated (C3, C5, CXCL2). Our finding is in partial consistent with the studies reported by Breijo, who have shown that the complement was activated during early secondary *E*. *granulosus* infection in mice [32]. It is probably due to the reason that when *E*. *granulosus* invaded, the hydatid cyst established is potentially able to activate host complement [32], resulting in the upregulation of C3a release, which attract and active inflammatory cells, thereby releasing the proinflammation cytokines, as suggested by the upregulation of CXCL2 chemokines. It has been known that various complement cleavage products have effector functions. C3 ligate their seven transmembrane-spanning G protein-coupled receptors C3aR, transmitting proinflammatory signals that induce vasodilation and cytokine and chemokine release [30].

As expected, *E*. *granulosus* infection at 8wks witnessed a phenomenon that expression of genes associated with vasodilation changed in the present study. Angiotensin converting enzyme (ACE) 2, a recently identified homologue of ACE that may regulate the actions of angiotensin (Ang) II by facilitating its breakdown to Ang 1–7, is found to be positively associated with vasodilation. It has been reported that in chronic liver injury, ACE2 is significantly up-regulated [33], showing the consistency with our study. Furthermore, we have shown that expression profiles of genes associated with inflammatory hyperemia in the blood metabolism (HBB, HBG) and muscle contraction were significantly altered (SNTB1, Gabrb2, Pou2af1).

In short, from the above phenomenon, we concluded that it may be due to the inflammation effect mediated by complement, as the complement was a key trigger of innate inflammation in *E*. *granulosus* infection. At 8wks, the larval stage or metacestode of *E*. *granulosus* (hydatid cyst) causes a chronic infection, the complement involved in the immune system was activated, which attract and activate inflammatory cells, thereby releasing the chemoattractants CXCL2, thus initiate an early phase of neutrophil recruitment. In the process of fighting against the parasite, these cells release histamine and other biologically active molecular, thereby causing blood vessels to dilate capillaries through increased permeability, smooth muscle contraction, thus mediate local inflammation [30, 32, 34]. Our results are in partial agreements with reports on *E*. *granulosus* infection in mice, showing that the hydatid cyst establishment are associated with the control of complement system and, consequently, of local inflammation at the early stages of infection [32].

However, we found the down-regulation genes encoding proinflammation cytokines (e.g., CCR6, CXCR6), which may be related to the cross-talk between the parasite and host. The control of host inflammation by the hydatid cyst most likely is achieved by inhibiting the formation and/or activity of a whole range of host pro-inflammatory.

On the other hand, we give the explanation that phagocytosis might be critical in complement mediated fighting against parasites at 8wks post infection.

In our study, we identified a marked up-regulation of several genes that are associated with mitochondrial metabolism (AACS, ND1, MCM, and DARS) in the liver of *E*. *granulosus* infection sheep at week 8. (ND1 gene encodes NADH dehydrogenase subunit 1 which is in the complex I of the respiratory chain. NADH1 is responsible for the energy generation pathway via producing ATP. MCM is involved in gluconeogenesis, in ruminants [35]. All of the above mentioned genes are involved in the mitochondrial energy metabolism process. As a central hub of energy metabolism, mitochondrial, plays an important role in cellular energy metabolism, and play an important role in the interaction between parasites and their hosts [36].

Combined the up-regulation of complements observed in this study, we assumed it may be resulted from complement mediated phagocytosis mechanism, which play the critical role in fighting pathogens [37]. In our study, C6, C4a, CR2 molecules produced during the complement activation process are important opsonin, which are able to combine with the complement receptor on the surface of phagocytic cells, and thus promote the role of phagocytic. Once phagocyte pathogens, phagocytic cells show active aerobic metabolism, and the consumption of oxygen is significantly increased in a short time, which is the “outbreak of the respiratory chain” phenomenon, as suggested by the significantly up-regulation of genes involved in the mitochondrial respiratory chain (ND1, MCM, DARS) in this study, and then activate the oxidase located on the cell membrane and phagosome membrane—reduced coenzyme 1 (NADH) and also coenzyme 2 (NADPH), catalytic oxygen molecules, playing the killing effects. In the present study, the up-regulation of NADH1 might partially reflect this assumption.

Signal transduction is an essential process in integrating host immune responses to pathogen infection. In the parasite infection process, a set of proteins involved in such pathways were significantly regulated during immunity development [22]. We also observed significant upregulation of genes associated with transport and signaling altered in this study. This might be due to the reason that the products produced by the immune system in the process fighting against parasite need to be transported out of the cells.

Last but not least, an immune homeostasis was established by the host during the infection process.

It is well known that the liver plays an important role in homeostasis and the overall metabolism of animals. While a robust and rapid initiation of the host defense mechanism is essential for a successful parasite clearance during the early cyst established phase, on the other hand, an excessive immune defense can produce temporary or permanent damage of the host, as suggested by the up-regulation of genes associated with liver injury (PIK42B, GPR155 and FAIM2) in the present study. Therefore, a restriction of a thriving innate immune response is necessary to limit host defense[38].

Here, we observed a strong down-regulation of genes that are associated with antigen presenting cells (MHC, CIITA, CD1, CD3, CD4, CD28), antibody (IGSF3, IGSF5) in CE infection sheep at 8wks after the experimental challenge. *In vitro*, it has been shown that complement and antibody together act as potential antiparasitic effector to inhibit parasite growth or kill metacestode [2].However in our study, we only observed the complement and its mediated effect was activated in parasite control, while antibodies and APC cells were repressed. This might be due to the reason that down-regulation of these genes balances the host immune response. However, there are also some questions need to be answered, firstly, whether repression of the antibodies and APC cells represents a time-dependent response, i.e, whether the antibodies and APC cells could be activated or remain this repressed expression level in the late stage of cyst established. On the other hand, it is likely that a more distal tissue from central parasitic vesicle might have a different degree of expression. Therefore, gene expression profiles in the lesion, including the periparasitic infiltrate, and in the surrounding liver, close to the lesions, were also needed to carry out. As mentioned above, we will design new studies respecting temporal and also spatial aspects to unravel late stage characteristics of CE in sheep.

## Conclusion

In summary, we have identified, for the first time, the gene expression pattern induced for *E*. *granulosus* infection at the liver tissue using a microarray-based methodology, resulting in identification of genes associated with immunity, liver injury as well as those implicated in the metabolism, signaling and transport pathways. Together with the previous studies, data are suggestive of the immunity, with the aid of metabolism system, and signalling pathways, plays crucial roles in the *E*. *granulosus* infection. However, as potential limitations in our study may influences gene expression, such as the small amount of sample size and the cellular composition tissue used, etc. Further studies-like amplifying the sample size, separating different cell types from the liver tissue, together with histology-should be carried out to confirm this hypothesis. And also, the proteomic analysis should be performed to examine gene transcription.

## Supporting Information

S1 Fig The morphology of hepatic parasitic lesions after 8 weeks infection in sheep. (TIF)

## References

[1] Eckert J, Deplazes P. Biological, epidemiological, and clinical aspects of echinococcosis, a zoonosis of increasing concern. Clin Microbiol Rev. 2004; 17: 107–135. 14726458 10.1128/CMR.17.1.107-135.2004 PMC321468

[2] Zhang W, Li J, McManus DP. Concepts in immunology and diagnosis of hydatid disease. Clin Microbiol Rev. 2003; 16:18–36. 12525423 10.1128/CMR.16.1.18-36.2003 PMC145297

[3] McManus DP, Zhang W, Li J, Bartley PB. Echinococcosis. Lancet. 2003; 362(9392):1295–304. 14575976 10.1016/S0140-6736(03)14573-4

[4] Brunetti E, Kern P, Vuitton DA. Expert consensus for the diagnosis and treatment of cystic and alveolar echinococcosis in humans. Acta Trop. 2010; 114: 1–16. 10.1016/j.actatropica.2009.11.001 19931502

[5] Torgerson P. Economic effects of echinococcosis. Acta Trop. 2003; 85: 113–118. 12606088 10.1016/s0001-706x(02)00228-0

[6] Cardona GA, Carmena D. A review of the global prevalence, molecular epidemiology and economics of cystic echinococcosis in production animals. Vet Parasitol. 2013; 192: 10–32. 10.1016/j.vetpar.2012.09.027 23084536

[7] Zhang W, You H, Li J, Zhang Z, Turson G, Aili H, et al Immunoglobulin profiles in a murine intermediate host model of resistance for *Echinococcus granulosus* infection. Parasite Immunol. 2003; 25:161–8. 12911524 10.1046/j.1365-3024.2003.00622.x

[8] Riganò R, Buttari B, De Falco E, Profumo E, Ortona E, Margutti P, et al *Echinococcus granulosus*-specific T-cell lines derived from patients at various clinical stages of cystic echinococcosis. Parasite Immunol. 2004; 26:45–52. 15198645 10.1111/j.0141-9838.2004.00682.x

[9] Amri M, Aissa SA, Belguendouz H, Mezioug D, Touil-Boukoffa C. In vitro antihydatic action of IFN-gamma is dependent on the nitric oxide pathway. J Interferon Cytokine Res. 2007; 27: 781–7. 17892399 10.1089/jir.2007.0003

[10] Zeghir-Bouteldja R, Amri M, Aitaissa S, Bouaziz S, Mezioug D, Touil-Boukoffa C. In Vitro Study of Nitric Oxide Metabolites Effects on Human Hydatid of Echinococcus granulosus. J Parasitol Res. 2009; 2009: 624919 10.1155/2009/624919 20798753 PMC2925086

[11] Mourglia-Ettlin G, Marqués JM, Chabalgoity JA, Dematteis S. Early peritoneal immune response during *Echinococcus granulosus* establishment displays a biphasic behavior. PLoS Negl Trop Dis. 2011; 5(8):e1293 10.1371/journal.pntd.0001293 21912714 PMC3166041

[12] Zhang W, Wen H, Li J, Lin R, McManus DP. Immunology and immunodiagnosis of cystic echinococcosis: an update. Clin Dev Immunol. 2012; 2012:101895 10.1155/2012/101895 22235225 PMC3253442

[13] Wang H, Li J, Pu H, Hasan B, Ma J, Jones MK, et al *Echinococcus granulosus* infection reduces airway inflammation of mice likely through enhancing IL-10 and down-regulation of IL-5 and IL-17A. Parasit Vectors. 2014; 20:522. 10.1186/s13071-014-0522-6 PMC4256745 25409540

[14] Wang Y, Wang Q, Lv S, Zhang S. Different protein of *Echinococcus granulosus* stimulates dendritic induced immune response. Parasitology. 2015; 142:879–89. 10.1017/S0031182014002005 25711466

[15] Virginio VG, Monteiro KM, Drumond F, de Carvalho MO, Vargas DM, Zaha A, et al Excretory/secretory products from in vitro-cultured *Echinococcus granulosus* protoscoleces. Mol Biochem Parasitol. 2012; 183:15–22. 10.1016/j.molbiopara.2012.01.001 22261090

[16] Siracusano A, Riganò R, Ortona E, Profumo E, Margutti P, et al Immunomodulatory mechanisms during *Echinococcus granulosus* infection. Exp Parasitol. 2008; 119:483–9. 10.1016/j.exppara.2008.01.016 18329023

[17] Vatankhah A, Halász J, Piurkó V, Barbai T, Rásó E, Tímár J. Characterization of the inflammatory cell infiltrate and expression of costimulatory molecules in chronic *echinococcus granulosus* infection of the human liver. BMC Infect Dis. 2015; 15:530 10.1186/s12879-015-1252-x 26578348 PMC4647452

[18] Mourglia-Ettlin G, Cucher M, Arbildi P, Rosenzvit M, Dematteis S. Natural and induced antibodies contribute to differential susceptibility to secondary cystic echinococcosis of Balb/c and C57Bl/6 mice. Immunobiology. 2016; 221:103–15. 10.1016/j.imbio.2015.07.016 26238549

[19] McManus D. Immunodiagnosis of sheep infections with *Echinococcus granulosus*: in 35 years where have we come? Parasite Immunol. 2014; 36: 125–130. 10.1111/pim.12072 24033483

[20] Hui W, Jiang S, Tang J, Hou H, Chen S, Jia B, Ban Q. An Immediate Innate Immune Response Occurred In the Early Stage of E. granulosus Eggs Infection in Sheep: Evidence from Microarray Analysis. PLoS One. 2015; 10(8):e0135096 10.1371/journal.pone.0135096 26252489 PMC4529311

[21] Gottstein B, Wittwer M, Schild M, Merli M, Leib SL, Müller N, et al Hepatic gene expression profile in mice perorally infected with *Echinococcus multilocularis* eggs. PloS one. 2010; 5: e9779 10.1371/journal.pone.0009779 20368974 PMC2848562

[22] Lin R, Lü G, Wang J, Zhang C, Xie W, Lu X, et al Time course of gene expression profiling in the liver of experimental mice infected with *Echinococcus multilocularis*. PloS one. 2011; 6: e14557 10.1371/journal.pone.0014557 21283804 PMC3023716

[23] Wang J, Lin R, Zhang W, Li L, Gottstein B, Blagosklonov O, et al transcriptional profiles of cytokine/chemokine factors of immune cell-homing to the parasitic lesions: a comprehensive one-year course study in the liver of *E*. *multilocularis*-infected mice. PloS One. 2014; 9: e91638 10.1371/journal.pone.0091638 24637903 PMC3956718

[24] Tsai IJ, Zarowiecki M, Holroyd N, Garciarrubio A, Sanchez-Flores A, Brooks KL, et al The genomes of four tapeworm species reveal adaptations to parasitism. Nature. 2013; 496: 57–63. 10.1038/nature12031 23485966 PMC3964345

[25] Zheng H, Zhang W, Zhang L, Zhang Z, Li J, Lu G, et al The genome of the hydatid tapeworm *Echinococcus granulosus*. Nat Genet. 2013; 45: 1168–1175. 10.1038/ng.2757 24013640

[26] Koziol U, Brehm K. Recent advances in Echinococcus genomics and stem cell research. Vet Parasitol. 2015; 213(3–4):92–102. 10.1016/j.vetpar.2015.07.031 26296590

[27] Yu H, Jia B. Tracking and monitoring the pathological processes of cystic echinococcosis in Kazakh sheep. Anim Sci Vet Med. 2012; S1:362.

[28] Rogan MT, Bodell AJ, Craig PS. Post-encystment/established immunity in cystic echinococcosis: is it really that simple? Parasite immunol. 2015; 37: 1–9. 10.1111/pim.12149 25283301

[29] Mathern DR, Heeger PS. Molecules Great and Small: The Complement System. Clin J Am Soc Nep. 2015;10: 1636–50. 10.2215/CJN.06230614 PMC4559511 25568220

[30] Swe PM, Reynolds SL, Fischer K. Parasitic scabies mites and associated bacteria joining forces against host complement defence. Parasite Immunol. 2014; 36:585–93. 10.1111/pim.12133 25081184

[31] Triantafilou M, Hughes TR, Morgan BP, Triantafilou K. Complementing the inflammasome. Immunology. 2016; 147:152–64. 10.1111/imm.12556 26572245 PMC4717242

[32] Breijo M, Anesetti G, Martínez L, Sim RB, Ferreira AM. *Echinococcus granulosus*: the establishment of the metacestode is associated with control of complement-mediated early inflammation. Exp Parasitol. 2008; 118:188–96. 17905232 10.1016/j.exppara.2007.07.014

[33] Paizis G, Tikellis C, Cooper ME, Schembri JM, Lew RA, Smith AI, et al Chronic liver injury in rats and humans upregulates the novel enzyme angiotensin converting enzyme 2 Gut. 2005; 54: 1790–1796. 16166274 10.1136/gut.2004.062398 PMC1774784

[34] He W, Cao XT, Xiong SD, Gao XM. Medical immunology 2^nd^ ed. Beijing: People’s Medical Publishing House; 2010 (In Chinese).

[35] Kennedy DG, Kennedy S, Blanchflower WJ, Scott JM, Weir DG, Molloy AM, et al Cobalt-vitamin B12 deficiency causes accumulation of odd numbered, branched-chain fatty acids in the tissues of sheep.Br J Nutr.1994;71:67–76. 7906141 10.1079/bjn19940111

[36] Labaude S, Cézilly F, Tercier X, Rigaud T. Influence of host nutritional condition on post-infection traits in the association between the manipulative acanthocephalan Pomphorhynchus laevis and the amphipod Gammarus pulex. Parasite Vectors. 2015; 8:403 10.1186/s13071-015-1017-9 PMC4520090 26223476

[37] Li E, Tako EA, Singer SM. Complement Activation by Giardia Parasites through the Lectin Pathway Contributes to Mast Cell Responses and Parasite Control. Infect Immun. 2016; 1:74–16. 10.1128/IAI.00074-16 PMC4807472 26831470

[38] Robert MA, Laura IR, Joseph FU, Miguel JS, William CG. Protective immune mechanisms in helminth infection. Nat Rev Immunol. 2007; 7: 975–987. 18007680 10.1038/nri2199 PMC2258092

