# Supplementary figures and images for: Gene Expression Profile in the Liver of Sheep Infected with Cystic Echinococcosis
Source: PLoS One. 2016 Jul 28;11(7):e0160000. doi: 10.1371/journal.pone.0160000 (PMC4965101; doi:10.1371/journal.pone.0160000)

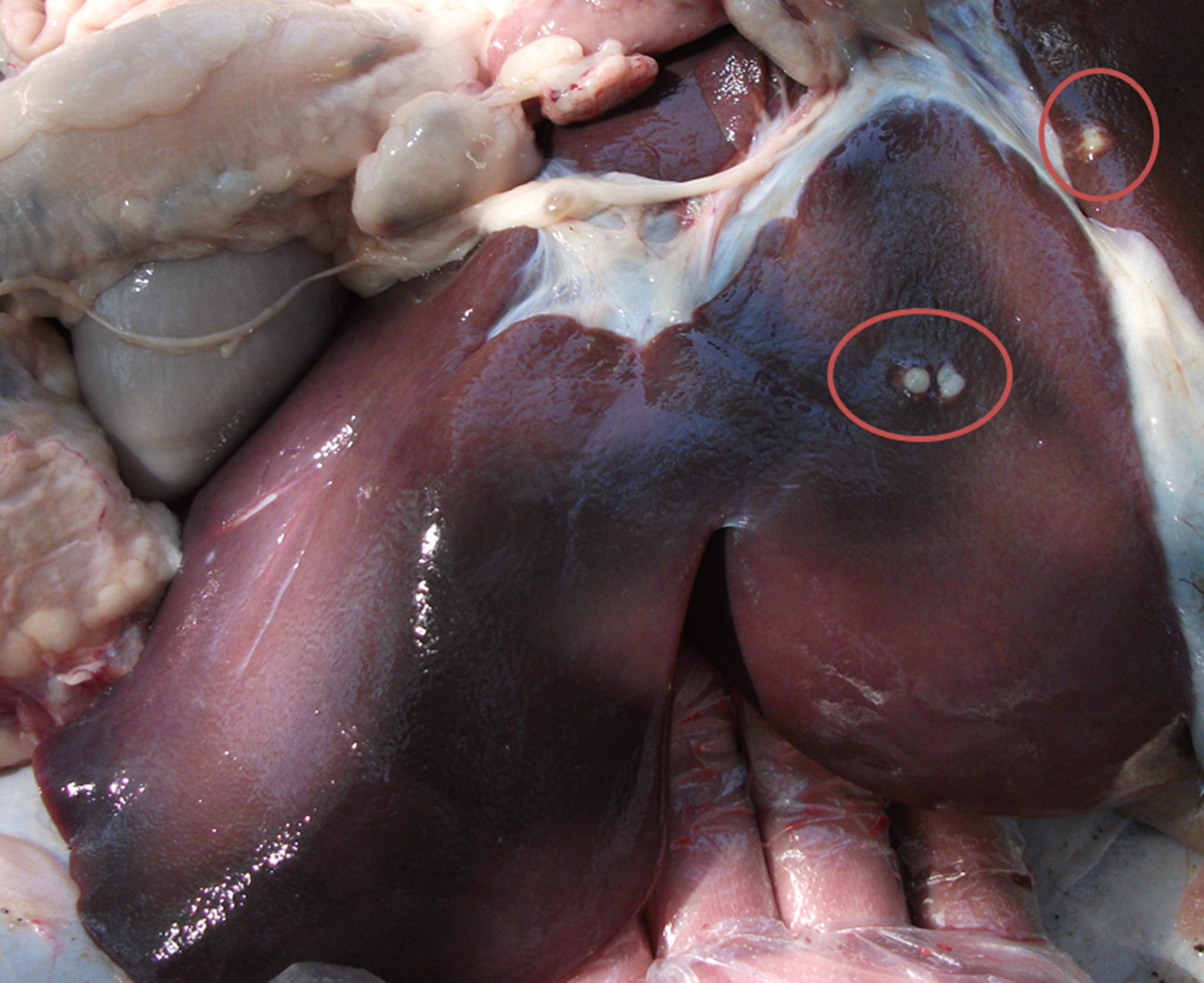

Supplement: S1 Fig — (TIF) [file pone.0160000.s001.tif]
